# Neurobehavioral and Biochemical Evidences in Support of Protective Effect of Marrubiin (Furan Labdane Diterpene) from *Marrubium vulgare* Linn. and Its Extracts after Traumatic Brain Injury in Experimental Mice

**DOI:** 10.1155/2022/4457973

**Published:** 2022-05-24

**Authors:** Nidhi, Govind Singh, Rekha Valecha, Govind Shukla, Deepak Kaushik, Mohammad Akhlaquer Rahman, Rupesh K. Gautam, Kumud Madan, Vineet Mittal, Rajeev K. Singla

**Affiliations:** ^1^Department of Pharmaceutical Sciences, Maharshi Dayanand University, Rohtak 124001, India; ^2^Department of Pharmacy, Delhi Skill and Entrepreneurship University, New Delhi 110065, India; ^3^University College of Ayurveda, Dr. S. R. Rajasthan Ayurveda University, Jodhpur 342304, India; ^4^Department of Pharmaceutics and Industrial Pharmacy, College of Pharmacy, Taif University, P.O. Box 11099, Taif 21944, Saudi Arabia; ^5^Department of Pharmacology, MM School of Pharmacy, MM University, Sadopur-Ambala 134007, India; ^6^Lloyd Institute of Management and Technology (Pharm.), Greater Noida, India; ^7^Institutes for Systems Genetics, Frontiers Science Center for Disease-Related Molecular Network, West China Hospital, Sichuan University, Chengdu 610041, Sichuan, China; ^8^iGlobal Research and Publishing Foundation, New Delhi 110059, India

## Abstract

Traumatic brain injuries due to sudden accidents cause major physical and mental health problems and are one of the main reasons behind the mortality and disability of patients. Research on alternate natural sources could be a boon for the rehabilitation of poor TBI patients. The literature indicates the *Marrubium vulgare* Linn. and its secondary metabolite marrubiin (furan labdane diterpene) possess various pharmacological properties such as vasorelaxant, calcium channel blocker, antioxidant, and antiedematogenic activities. Hence, in the present research, both marrubiin and hydroalcoholic extracts of the plant were evaluated for their neuroprotective effect after TBI. The neurological severity score and oxidative stress parameters are significantly altered by the test samples. Moreover, the neurotransmitter analysis indicated a significant change in GABA and glutamate. The histopathological study also supported the observed results. The improved neuroprotective potential of the extract could be attributed to the presence of a large number of secondary metabolites including marrubiin.

## 1. Introduction

Injuries are a major public health problem today. Traumatic brain injury (TBI) is defined as damage to the brain resulting from an external mechanical force, such as that caused by rapid acceleration or deceleration, blast waves, crush, or impact, and can lead to temporary or permanent impairment of cognitive, physical, and psychosocial functions [1–3]. TBIs in India have been increasing significantly due to rapid motorization, industrialization, migration, and changing value systems of Indian society. Also, the Centre for Disease Control and Prevention estimated that the incidence of TBI affects a large number of the population worldwide. Apart from instantaneous deaths, the suffering and poor quality of life among survivors is a living testimony to the impact of TBIs. The total volume of TBI in India is unknown, but estimates suggest that there are more than a million trauma-related deaths in India per year, of which 50% are TBI-related [4, 5]. It involves a complex cascade of changes including pathological, metabolic, and gene-related changes which not only include cell damage but also contribute to the activation of inflammatory cytokines (IL-6, TNF-*α*), increase in intracellular calcium regulation, oxidative stress, and mitochondrial dysfunction that contributes to neurodegeneration. Other pathological changes as part of secondary injury include the neurological deficit, activation of microglial cells, disruption of the blood-brain barrier, cerebral edema, intestinal damage, and abnormal nitrite, glutamate, and calcium level [6–8]. The study of the literature indicated that there is not any particular treatment strategy for TBI and research on new compounds or plant metabolites is highly required to improve the quality of life [9]. Enough pieces of evidence are present in the literature which could prove the phytochemicals/extracts to be effective and protective in TBI. A huge number of herbal extracts or plant actives are also reported to possess a significant role in neuroprotection, reduction of inflammation, and management of oxidative stress [10–14].

The *Marrubium* genus (Family: Lamiaceae) has nearly thirty plant species and is indigenous to Europe and Asian countries. Among various species, *Marrubium vulgare* L. is a perennial herb commonly known as “white horehound” in different regions of Europe [15]. The hydroalcoholic extract of selected medicinal herbs was reported to possess significant pharmacological properties by inhibiting the action of neurotransmitters such as acetylcholine, prostaglandin E, histamine, and bradykinin [16–19]. The various secondary metabolites of different categories such as marrubiin (diterpene), arenarioside, acteoside, forsythoside B, and ballotetroside (phenylpropanoids esters) have been isolated and identified from the plant extracts [20–24]. The pharmacological potential of marrubiin as anti-inflammatory, vasorelaxant, antioxidant, and calcium channel (L-type) blocker has been well established [25]. The other diterpenes present in the plant extract like marrubinic acid and marrubenol also exhibited analgesic and antiedematogenic activities [23]. Moreover, the various phenylpropanoid esters of the herb showed significant anti-inflammatory potential due to the inhibition of the cyclooxygenase-2 (COX-2) enzyme [22].

**Figure 1:**
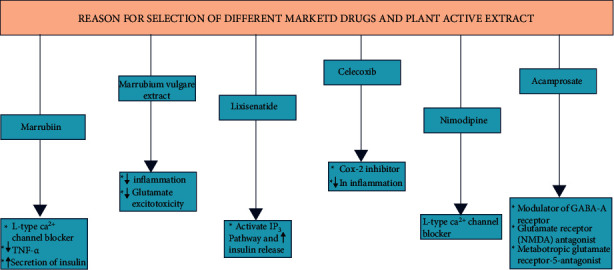
Pharmacological rationale for the selection of various test samples (plant active/extract) and marketed drugs for the present study.

On the basis of a literature study, the hydroalcoholic extract of *Marrubium vulgare* L. and marrubiin, a potential and well-tolerated diterpene (LD_50_ 370 mg/kg) of the herb, are selected to evaluate their protective effect against TBI in experimental mice. Further, the research work is also extended to evaluate the possible synergistic protective effect of plant active (marrubiin)/extract with some selected marketed drugs like lixisenatide (L), nimodipine (N), acamprosate (A), and celecoxib (C) (Figure 1) [12, 26, 27].

## 2. Results and Discussion

The selected medicinal plant, *Marrubium vulgare* L., and its active constituent, marrubiin, are reported to possess numerous pharmacological properties such as vasorelaxant, antioxidant, antihypertensive, antispasmodic, anti-inflammatory, antiedematogenic, and antidiabetic properties [17, 25]. Hence, in the present study, the plant active and hydroalcoholic extract of the herb is explored for its neuroprotective potential against TBI. Moreover, the selected dose of plant extract/active is also investigated for protective action in combination with some marketed drugs like lixisenatide, celecoxib, nimodipine, and acamprosate.

The powdered sample of the plant was extracted by cold maceration method and a dark brown extract was obtained with a percentage yield of 8.5 ± 1.2% (w/w). The extract was also analyzed by HPTLC for marrubiin concentration and was calculated to be present in 7.1 ± 0.8% (w/w) concentration. On the basis of literature and the result of extract analysis, two doses of the extract, 700 mg/kg and 1400 mg/kg, were selected for the present experimental protocol. The Swiss albino mice were used to study the neuroprotective effect of the herb and its active principle along with selected marketed drugs. TBI was induced in the animals of different groups, except control, by the weight drop method.

### 2.1. Assessment of Neurological Severity Score

**Figure 2:**
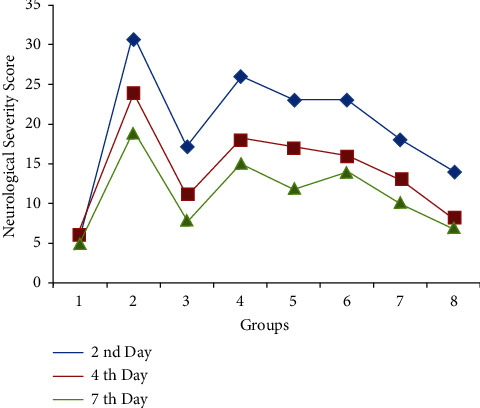
Change in the neurological severity score on different days of experimental study (2^nd^, 4^th^, and 7^th^ day).

After TBI, the assessment of NSS reflects the rough magnitude of motor and cognitive deficiency such as difficulty in memory, attention deficits, and decrease in concentration in affected animals. Early assessment of NSS is a simple and reliable method to evaluate the motor ability and cognitive skills in injured rodents. Numbers of tasks were performed to check the NSS like straight walk, exit circle, grip strength, startle reflex, beam walk, and round stick balance. NSS of 5–7 indicated moderate injury in the experimental animals of negative control (group 2) [28–30]. The NSS of animals from different groups was presented in Figure 2.

The interpretation of data confirmed that plant extracts at 1400 mg/kg significantly altered the score in the treated group as compared to the −ve control after the 7^th^ day of treatment protocol (*p* < 0.05). Further, this extract in combination with selected marketed drugs (Group 8) significantly reduced the severity score even after the 4^th^ and 7^th^ days of treatment (*p* < 0.01). The assessment of NSS gives a fair idea about the neuroprotective effect of plant extract alone and in combination with marketed drugs. Further, the motor coordination, mobility, and memory potential in trauma-affected animals were evaluated by performance in the open field, rotarod, and plus-maze activities.

#### 2.1.1. Open Field Performance Analysis

**Figure 3:**
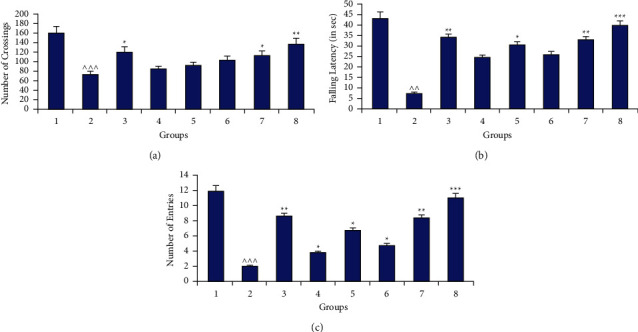
Effect of different neurobehavioral tests: (a) open field, (b) rotarod, and (c) elevated plus maze in different groups (1: control; 2: (−ve) control (TBI); 3: marketed drugs (L + C + N + A); 4, 5: marrubiin (50,100 mg/kg); 6, 7: *M. vulgare* extract (700,1400 mg kg); 8: *M. vulgare* extract (1400 mg/kg) + marketed drugs) (^∧^*p* < 0.05; ^∧∧^*p* < 0.01; ^∧∧∧^*p* < 0.001 as compared to the control group; ^*∗*^*p* < 0.05; ^*∗∗*^*p* < 0.01; ^*∗∗∗*^*p* < 0.001 as compared to the −ve control).

On the 7^th^ day of the experimental protocol, the mobility/locomotor performance in various animals was further analyzed by open field and rotarod performance analysis. In this test, the number of lines crossed by mice from different groups was counted for at least 5 minutes and presented graphically in Figure 3(a). Results confirmed that the trauma significantly (*p* < 0.001) reduced the number of lines crossed by mice (77 ± 6.4) as compared to the control group (160 ± 12.4). Further, the animals treated with plant extract (1400 mg/kg) enhanced their mobility (*p* < 0.05) which was further improved significantly when this extract was supplemented with marketed drugs (*p* < 0.01).

#### 2.1.2. Rotarod Performance Analysis

Motor coordination in the experimental animals was further investigated by analysis of rotarod performance. In this experiment, the falling latency of animals of various groups from a rotating rod (25 rpm) for five minutes was observed and noted (Figure 3(b)). Falling latency in the control group (43 ± 2.510) was significantly (*p* < 0.01) reduced in the TBI-affected group (7.6 ± 1.631). Delay in falls was also altered in animals treated with marrubiin at different doses but the change was not significant as compared to the affected group (*p* > 0.05). Moreover, the extract of the selected medicinal plant at a higher dose significantly enhanced the falling latency of experimental animals (*p* < 0.01). The significance of the result was further improved when the selected dose (1400 mg/kg) of the extract was administered along with all marketed drugs (*p* < 0.001).

#### 2.1.3. Elevated Plus Maze Study

TBI can also cause the short-term loss in memory of the animals under the protocol. A simple apparatus such as an elevated plus maze could be employed to evaluate the cognitive function in different groups. On the seventh day of the experiment, the number of entries in the closed arm of the elevated plus maze was recorded for a minimum of 5 minutes (Figure 3(c)). Loss in memory potential of injured mice was indicated by the significant reduction (*p* < 0.001) in the number of entries (1.6 ± 0.32) as compared to the control group (12 ± 1.1). Treatment with plant extracts/active constituents and different drugs altered the memory deficit induced by the TBI. But the improvement in impaired memory function was significant in animals fed with plant extract at high doses (*p* < 0.01). Further, the synergistic neuroprotective effect was exhibited by rodents on giving the extract along with selected commercial drugs.

#### 2.1.4. Estimation of Biochemical Parameters

**Figure 4:**
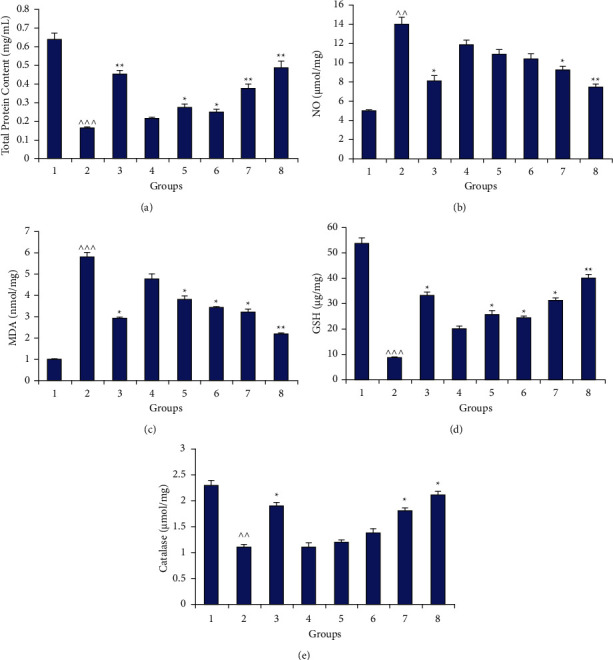
Evaluation of various biochemical (oxidative stress) parameters: (a) total protein content, (b) nitric oxide, (c) malondialdehyde (MDA), (d) glutathione (GSH), and (e) catalase, in different groups (1: control; 2: (−ve) control (TBI); 3: marketed drugs (L + C + N + A); 4, 5: marrubiin (50,100 mg/kg); 6, 7: *M. vulgare* extract (700,1400 mg kg); 8: *M. vulgare* extract (1400 mg/kg) + marketed drugs; ^∧^*p* < 0.05; ^∧∧^*p* < 0.01; ^∧∧∧^*p* < 0.001 as compared to the control group; ^*∗*^*p* < 0.05; ^∗∗^*p* < 0.01; ^*∗∗∗*^*p* < 0.001 as compared to the −ve control).

**Figure 5:**
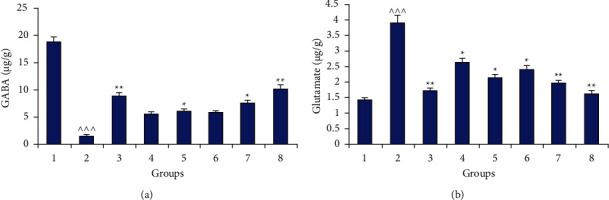
Neurotransmitter: (a) GABA (inhibitory) and (b) glutamate (excitatory) analysis in different groups (1: control; 2: (−ve) control (TBI); 3: marketed drugs (L + C + N + A); 4, 5: marrubiin (50,100 mg/kg); 6, 7: *M. vulgare* extract (700,1400 mg kg); 8: *M. vulgare* extract (1400 mg/kg) + marketed drugs; ^∧^*p* < 0.05; ^∧∧^*p* < 0.01; ^∧∧∧^*p* < 0.001 as compared to the control group; ^*∗*^*p* < 0.05; ^∗∗^*p* < 0.01; ^*∗∗∗*^*p* < 0.001 as compared to the −ve control).

**Figure 6:**
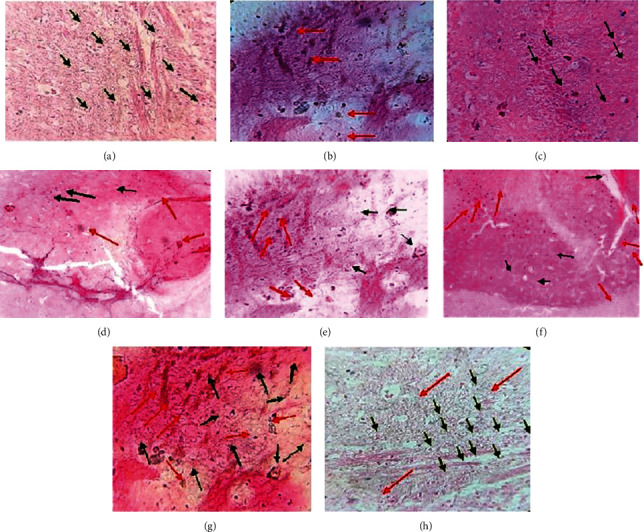
Photomicrographs of brain sections of experimental animals from different groups (a) control; (b) (−ve) control (TBI); (c) marketed drugs (L + C + N + A); (d, e) marrubiin (50,100 mg/kg); (f, g) *M. vulgare* extract (700,1400 mg kg); (h) *M. vulgare* extract (1400 mg/kg) + marketed drugs) depicting intact neuron by black arrow and nonintact cells by red arrow.

The concentration of various endogenous oxidative stress parameters (GSH, MDA, Catalase) was determined and was indicated in Figure 4. TBI significantly reduced the GSH concentration as compared to the control group (*p* < 0.001) whereas the level of malondialdehyde and catalase increased significantly (*p* < 0.01) (Figure 5). Further, the results suggested that the plant extract alone in higher concentration and along with the marketed drugs significantly altered the oxidative stress parameters (*p* < 0.05 and *p* < 0.01, respectively). Moreover, the total protein content in the test samples was determined and concentration was found to be significantly altered in treated groups (Figure 6). Also, the nitric oxide (NO) concentration was altered by test groups but the change was significant with the marrubiin (50 mg/kg) and plant extract at a higher dose (*p* < 0.05).

#### 2.1.5. Neurotransmitters (GABA and Glutamate) Analysis

The concentration of inhibitory (GABA) and excitatory (Glutamate) neurotransmitters was modulated significantly (*p* < 0.001) in the animals suffering from traumatic brain injury (Figure 6). Treatment with test drugs (extract and plant active) at different doses enhanced the GABA concentration but the change was insignificant (*p* > 0.05) with a low dose of marrubiin and plant extract. But a higher dose of the extract (*p* < 0.01) and active constituent (*p* < 0.05) of the herb displayed a significant change in the quantity of GABA. Further, the glutamate analysis confirmed the significant reduction (*p* < 0.01) in its level on treatment with *Marrubium vulgare* extract at an elevated dose (1400 mg/kg). Also, there is a synergistic alteration in the effect produced by herbal extract in combination with marketed drugs (*p* < 0.001).

#### 2.1.6. Histopathological Study

**Figure 7:**
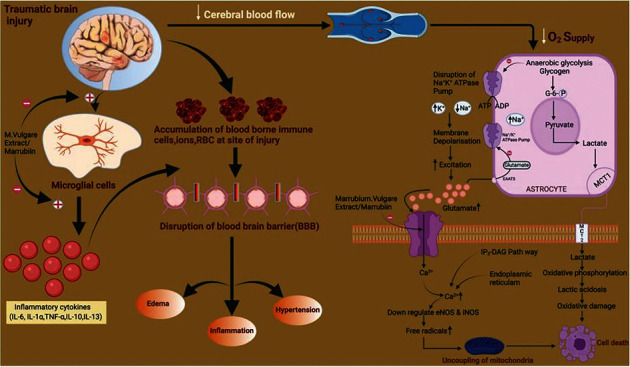
Proposed site of action of *Marrubium vulgare* extract/marrubiin to bring out the protective effect after TBI.

On the 8^th^ day of the experimental protocol, the histopathological changes in the cerebral cortex of brain sections from the animals of different treated groups were observed. The nuclei (pink stain) and cytoplasm (violet color) could be easily differentiated in the neuronal cell of brain tissue. The intact neuronal cell from the samples of different treated groups was depicted as a black arrow in the photographs, but when there is no clear distinction between cytoplasm and nuclei, then it was displayed as a red arrow in the section pictures (Figure 7).

Trauma due to accidents, blasts, and falls is the most prevalent cause of injury to the brain [31]. TBI not only causes the mortality of individuals but also produced a large number of short- and long-term consequences to brain functions. According to an estimate, TBI contributes to more than 30% of all injury-related deaths in the USA, and about half of the survivors (43%) faced significant chronic disabilities due to the nonavailability of clinically effective treatment protocol [32, 33]. After the TBI, there occur different primary and secondary pathological signaling cascades. The effect due to primary insult cannot be treated but can be prevented whereas the functional impairment and chronic disability of neurons after secondary injury because of oxidative stress, excitotoxicity, inflammation, and mitochondrial dysfunction could be targeted for the improvement of patient life after injury [8, 34].

The selected medicinal plant *Marrubium vulgare* L. and marrubiin (plant active) were investigated for neuroprotective effects after TBI. After TBI, the assessment of NSS reflects the rough magnitude of motor and cognitive deficiency such as difficulty in memory, attention deficits, and decrease in concentration in affected animals. Improvement in the motor ability and cognitive skills of injured rodents was exhibited by the various test groups especially the plant extract in a higher dose and its combination with marketed drugs. The literature revealed that marrubiin itself and the plant extract are potent calcium channel blockers and the significantly altered functional ability of mice might be due to this action [16, 35]. Also, the scientists had proved that the calcium channel blockers not only improved the behavioral deficits but also prevent the disruption in spatial memory of experimental animals [36, 37].

Moreover, sudden trauma to the brain triggers the different signaling pathways in astrocytes and neurons that lead to the upregulation of calcium and downregulation of endothelial nitric oxide synthase (eNOS). It further enhanced the concentration of reactive oxidative species (ROS) and leads to mitochondrial dysfunction and apoptosis [38, 39]. It has also been reported that NO, which is a unique molecule, causes cytotoxicity due to enhanced oxidative stress, and the plant extract significantly altered the various stress parameters along with the NO to exert the neuroprotective effect in the injured animals [40]. The observed results could be attributed to the blockage of Cav 1.2 and Cav 1.3 (L-type) postsynaptic calcium channels localized in the soma, spines, and shaft of dendrites which further help in the downregulation of enhanced calcium inflow and decrease the ROS production which could help in the restoration of the mitochondrial respiratory chain and its integrity [16, 35, 37]. In past, the treatment with plant extract was also postulated to reduce the microglial activation which could be another reason for better neuroprotective action by affecting the inflammatory cascade after TBI [18, 41, 42].

The results of the neurotransmitters analysis indicated that there is a significant alteration in GABA (↓) and glutamate levels (↑) after the injury. These findings match with previous investigations which enhanced the glutamate level and excitotoxicity induced signaling cascades which resulted in neurodegeneration [43]. Also, it has been postulated that the imbalance between these neurotransmitters leads to neurodegeneration in the brain [44–46]. But treatment with *Marrubium vulgare* extract and marrubiin significantly modulates their concentration in a positive manner. The neuroprotective effect of the test samples implies that excitotoxicity due to enhanced glutamate was reduced and the balance between GABA and glutamate was improved to bring out this action. Further, the calcium channel blockers, especially of L-type, were reported to reduce the necrosis in glutamate toxicity; therefore, the present results could be attributed to the inhibition of voltage-gated cation (Ca^2+^) channels by plant active and extract [16, 35, 47]. Also, the vasospasm is the obvious feature in a significant number of traumatic cases and vasorelaxant action of the plant active/extract could further enhance the recovery in the victims after injury [33, 48]. Moreover, the photomicrographs of the histopathology of brain sections further confirmed the neuroprotective nature of plant extract at a higher dose and its combination with selected marketed/commercial drugs.

The present findings indicated that recovery after the injury was more significant with the herb extract as compared to plant active alone. Such results could be due to the presence of pharmacologically important secondary metabolites including marrubiin, in the plant extract. Marrubiin was reported to block the L-type calcium channels and thus bring out the vasorelaxant and antiexcitotoxicity effect of herb. Also, the inhibition of these cation channels could reduce the activation of resident microglial cells and thus lessen the release of proinflammatory cytokines (IL- 1*α*, IL-6) [18, 41, 42]. Further, the previous investigations established that the different phytochemicals such as renarioside, acteoside, forsythoside B, and ballotetroside as phenylpropanoid esters present in the extract can inhibit the cyclooxygenase-2 (COX-2) enzymes and thus are responsible for the anti-inflammatory action of herb (C) [21, 24, 49]. Moreover, the hydroalcoholic extract was also reported to reduce the spasm induced by acetylcholine and oxytocin at experimental conditions [50]. In addition, oxidative stress was considered to be as one of significant parameters in inducing the secondary effects after TBI [51]. The level of GSH was reduced whereas the MDA and catalase concentration enhanced significantly after the injury in experimental animals. Treatment with the plant extract at higher dose significantly altered the oxidative stress parameters. The antioxidant potential of the herbal extract could be due to the presence of total phenolic and flavonoid content in addition to marrubiin which could also be a possible reason behind the fast recovery of injured animals [17, 52–54]. Finally, TBI also reported to induce the oedema in the brain which is mainly responsible for the significant number of mortality in the head injury cases and the selected plant extract/marrubiin also possess the antioedematogenic properties [23, 55]. Hence, the improved neuroprotective effect of the selected medicinal plant could also be attributed to the antioedematogenic potential along with other discussed properties. On the basis of the above discussion, the proposed site of action of plant extract/active to bring out the protective effect in the pathological cascade of TBI is depicted in Figure 7.

## 3. Materials and Methods

### 3.1. Plant Sample and Reagents

The selected herb, *Marrubium vulgare* L., was collected from the local district of Nainital, Uttarakhand, and identified by a botanist. A voucher specimen was also kept in the laboratory of the department for future reference. All the procured chemicals and reagents were of analytical grade. The plant active, marrubiin, was purchased from the reliable commercial source, Extrasynthese (France).

### 3.2. Extraction of Plant Sample and Analysis

The collected sample of the whole plant was thoroughly washed in running tap water and shade dried. The dried herb was powdered, sieved (60–80), and extracted with hydroalcoholic solvent (50%) by the cold maceration method. The powdered sample (100 g) was kept in a round bottom flask with solvent (1 : 15) at room temperature for 10 days with occasional stirring. The extract obtained was concentrated, lyophilized, and stored at low temperature in an airtight container till further study. Also, the qualitative and quantitative analysis of the extract for the marrubiin concentration was performed using a validated HPTLC protocol which was previously developed in our laboratory [56, 57].

### 3.3. Animals

For the present research, the swiss albino mice (25–30 g) were procured from the disease-free small animal house of Lala Lajpat Rai University of Veterinary and Animal Sciences (LLRUVAS), Hisar, India. The animals were housed in the group of five (*n* = 5) in polypropylene cages (29 × 22 × 14 cm) lined with proper bedding. They were acclimatized as per the standard CPCSEA guidelines for at least two weeks under natural light/dark cycle at 25 ± 2°C. During this period, they have free access to standard rodent feed and water *ad libitum*.

### 3.4. Experimental Protocol and Induction of Traumatic Brain Injury

**Figure 8:**
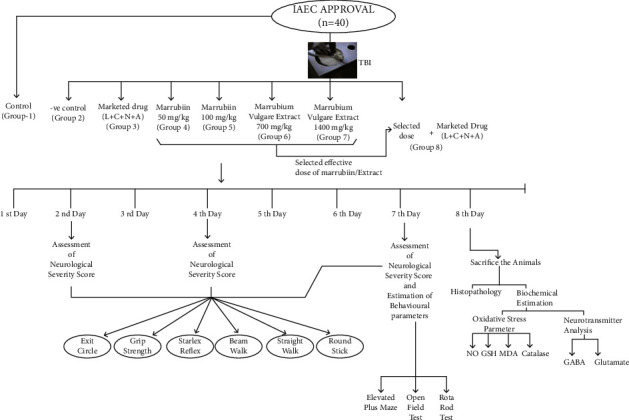
Detailed experimental protocol for the present study.

TBI is followed by not only the immediate primary effects but also some cellular, genomic, and biochemical changes termed the secondary insult. These effects could last up to some minutes to some days [58]. Hence, the experimental protocol should be kept for seven days to effectively evaluate the protective effect of the selected plant active/extract against secondary insult after TBI. Prior to the commencement of experiments, the research protocol was duly approved by the institutional animal ethical committee (1767/RE/S/14/CPCSEA/CAH/153–165, dated-17/12/18), MDU, Rohtak. After the specified time period (15 days) of acclimatization of experimental animals, they were divided into twelve groups (*n* = 5). The detailed experimental protocol is illustrated in Figure 8. The TBI was induced in the animals of all groups except the first group (control) by the standard weight-drop method. Animals were placed in a closed chamber and anesthesia was induced with 4% isoflurane. After 5–7 minutes, the mouse was removed from the closed chamber and placed on the open circuit with 2% isoflurane maintenance. The reflexes were checked by pressing the hind paw and observing the eye movements. In the absence of reflexes, the mouse was placed on the foaming bed, and a midline incision was given over the head of mice and exposed the skull. The metallic disc was fixed over the exposed skull and placed properly under the metallic pipe (1 m long). After that, 66 g weight (spherical brass ball) was dropped through the upper end of the pipe to induce closed head injury. After brain trauma, the scalp was sutured, neosporin powder (GlaxoSmithKline Pharmaceuticals Ltd., Banglore, India) was applied over the scalp, and the mouse was returned to their cage for recovery [59]. The test drugs/extracts were given to animals of different groups as per the protocol for seven days. The neurological severity score for each group was calculated after 24 hrs, 4^th^ day, and 7^th^ day, and behavioral parameters were studied on the 7^th^ day of the experimental study. On the next day, the animals were sacrificed by cervical dislocation and used for histological and biochemical study.

### 3.5. Neurobehavioral Study

Immediate after the TBI, the pathological cascade is initiated and it reflects through impairment in motor coordination, balance, and locomotor dysfunction of animals. Also, due to impact, there could be a loss of memory in experimental mice. The magnitude of these parameters could be assessed as per the following protocols.

#### 3.5.1. Assessment of Neurological Severity Score

The neurological severity score (NSS) roughly determines the magnitude of loss in motor and locomotor function. The various tasks like exit circle, beam walk, round stick, straight walk, startle reflex, and grip strength after 24 hrs (2^nd^ day), 4^th^ day, and 7^th^ day of injury were performed to calculate it. One point was given for non/delayed performance and zero points for the completion of tasks. All the tasks were carried out as per the standard procedures. Briefly, in the exit circle, the mice were placed in the center of the circle and the time taken to exit the circular instrument was monitored for three minutes. In beam walk, the animal was kept on the wooden beam on an elevated surface (60 cm). On the other end, a box (20 × 25 × 24 cm) with a 10 cm opening was placed. The time taken by the mice to reach the box was noted and compared with the control for the calculation of scores. For grip strength, the mice were held up by the tail, and their paws were touched by the anatomical forceps. If the mouse grips the forceps, 0 points were counted, and on failure or performance with very low intensity, 1 point was added. In the straight walk test, the mouse was placed on a clear surface and observed for the walking pattern. The impaired gait pattern and failure to actively explore environs by the animal are credited for 1 point and a normal walk earns nil. In the round stick balance test, the ability of mice to balance over a round stick (Φ 5 mm) for 10 seconds was evaluated. The alertness of the mice and response to the sound of a hand clap was evaluated in the starlet reflex test [60].

#### 3.5.2. Open Field Test

After the injury, the motor and exploratory behavior of mice can be evaluated by this simple test. In this, the mice were placed in the middle of the apparatus (70 × 70 × 25 cm) and the number of squares crossed by at least the anterior paw of animals was recorded for 5 min by visual inspection [61].

#### 3.5.3. Rota Rod Test

The coordination in the motor skills can further be evaluated by this test. The ability of the animals to hold on to the accelerated rotating rod depicts the motor functions. In this test, the mice were placed on a rotating rod (5 rpm), and it was accelerated at a speed of 5 rpm every 40 seconds. The animals were kept on the rotating rod till the rod speed reached 25 rpm. The average latency in the fall of mice from the rod was noted for 5 minutes [62].

#### 3.5.4. Elevated Plus Maze

The learning and memory potential of the drug in the experimental mice can be evaluated by the previously described methods. In this test, a wooden plus maze with open and closed arms was kept in an elevated place (25 cm from the floor). The mice were placed on the open arm of the elevated plus maze, and no entries in the closed arm and open arm were recorded for 5 min [63].

### 3.6. Analysis of Biochemical Parameters

On the eighth day of the experimental study, the whole brain of animals from each group was isolated and cleaned with ice-cold normal saline. The brain samples were homogenized with phosphate buffer (pH 7.4) and immediately centrifuged at 2500 rpm for 15 min. The homogenate was used to analyze the various biochemical parameters.

#### 3.6.1. Total Protein Content

Alkaline copper solution 5 ml was added to 1 ml of brain tissue homogenate and allowed to stand for 10 minutes. Further, the 0.5 ml of diluted Folin's reagent (1 : 2) was added and mixed properly. After 30 minutes, the absorbance of the prepared solution was taken at 750 nm in a UV-Visible spectrophotometer. The total protein content was determined in mg/mL [64].

#### 3.6.2. Estimation of Catalase

The catalase assay of the different samples was performed by the method described by Sinha et al., [65]. In this, the small portion of brain homogenate (0.1 mL) was mixed with 0.1 mL of phosphate buffer (0.01 M, pH 7.4) and 0.4 ml of distilled water. Further, the addition of 0.5 ml of H_2_O_2_ (2 M) initiated the reaction. The mixture was incubated for 1 min at room temperature and the reaction was ended by mixing 2 ml of potassium dichromate-acetic acid reagent. The solution was kept for 15 minutes in a boiling water bath. On cooling the solution, the green color appeared and the absorbance was taken at 570 nm in a UV-Visible spectrophotometer. The concentration of catalase was expressed as *μ*moles/mg of protein [66].

#### 3.6.3. Estimation of Malondialdehyde (MDA)

Malondialdehyde is an indicator of lipid peroxidation and was estimated as previously described [67]. The supernatant of tissue homogenate was mixed with various reagents like acetic acid (1.5 ml, 20%), thiobarbituric acid (1.5 ml, 0.8%), and sodium dodecyl sulfate (0.2 ml, 8.1%). The mixture was heated at 100°C for 60 min and cooled. After cooling, 5 ml of n-butanol: pyridine (15 : 1 v/v) and distilled water (1 ml) were added with vigorous shaking. The solution was centrifuged at 4000 rpm for 10 min, and the separated organic layer was removed and absorbance was measured at 532 nm using a UV-Visible spectrophotometer (Shimadzu UV Spectrophotometer, UV-1800 Series, India). The amount of malondialdehyde present in the sample was expressed as nmol/mg protein.

#### 3.6.4. Nitric Oxide (NO) Assay

The accumulation of nitrite was assayed spectrophotometrically using a Greiss reagent. The supernatant (100 *μ*l) was mixed with an equal volume of Greiss reagent and kept for 10 min at room temperature. The absorbance of the reaction mixture was measured at 540 nm against a blank solution (distilled water). The concentration of NO in the sample was calculated by plotting the standard curve of sodium nitrite (5–30 *μ*mol/ml) and was expressed as *μ*moles of NO/mg protein [68].

#### 3.6.5. Measurement of Reduced Glutathione (GSH)

The reduced glutathione content in the tissue homogenate was measured by the previously described method using a UV-Visible spectrophotometer. The processed sample was mixed with an equal volume of sulfosalicylic acid (5%) and then centrifuged at 2000 rpm at 4°C for 10 min to separate out the proteins. Take the supernatant and add phosphate buffer (2 mL, pH 8.4), 5,5′-dithiobis (2-nitrobenzoic acid, (0.5 mL), and distilled water (0.4 ml) thoroughly and take the absorbance at 412 nm. The concentration of GSH in the sample was determined using a standard curve prepared with reference GSH (5–25 *μ*g/ml) and expressed as *μ*g of GSH/mg protein [17, 69].

#### 3.6.6. Estimation of GABA and Glutamate

The ϒ-aminobutyric acid (GABA) and glutamate concentration in the brain samples of various test groups were estimated using HPTLC. The spots of the test and standard samples were applied with the help of a Linomat 5 applicator (CAMAG, Switzerland) on precoated silica gel plates (20 × 10 cm). The chromatogram was developed with n-butanol: glacial acetic acid: water (5 : 3 : 2) as a mobile phase. The plate was sprayed with ninhydrin (0.2%) and dried in an oven at 60–65°C for 3-4 minutes to visualize desired compounds. The developed plate was scanned at 482 nm and the area under the curve (AUC) of various components was quantitatively analyzed with the WINCAT software [70, 71].

### 3.7. Statistical Analysis

The statistical analysis of the data was performed by Prism Graph pad (version 9) software. The data is represented as mean ± SD (standard deviation) and the statistical significance of the data obtained in the various tests was carried out by the analysis of variance (ANOVA) followed by the Tukey test. The different significant level of data as compared to the control (group 1) or negative control (group 2) is also represented in bar diagrams and *p* < 0.05 is considered significant.

### 3.8. Histopathological Study

On the 8^th^ day of the experimental study, the mice were sacrificed and some of the brain samples from each group were dissected for histopathological study. The tissue was fixed in Bouin's fixative for 72 h. After following all standard procedures, the tissues were embedded in wax blocks and trimming of tissue (7 *μ*m thickness) was done using a microtome. The tissue sections were stained with eosin and hematoxylin dyes and the counterstaining was done with Dibutylphthalate Polystyrene Xylene. The stained tissue sections were analyzed at 20 × under a light microscope [72].

## 4. Conclusion

Increased incidences of the TBI posed a great challenge in terms of the physical, mental, economical, and social well-being of the victims. The secondary insult modulates not only the various neurobehavioral functions required for normal motor and cognitive functions but also induced the neurotoxic cascades due to enhanced oxidative stress and causes cell death and necrosis. Even the long back after the injury, the survivors of TBI, remained at the risk of inflammatory and immune-mediated pathological cascade leading to apoptosis and sudden death. The literature study revealed that along with the other alternatives, several herbal products (decoctions, pills, extracts) were also used for the management of TBI. The selected medicinal plant, *Marrubium vulgare* L., is traditionally used as a decoction in the Jammu and Kashmir region for many ailments. The results of the present study indicated that herb extract can also be used as neuroprotective after a traumatic head injury. Further, it is suggested that after establishing the role of the herb in the reduction of proinflammatory cytokines, the plant could be used for the faster recovery and rehabilitation of victims after TBI. Moreover, the financial burden of the secondary treatment cost for the poor patients with TBI could also be reduced by the use of such herbs.

## Data Availability

The data related to the submitted manuscript could be readily available to the reader on request.
